# Real World Performance Evaluation of Transcatheter Aortic Valve Implantation

**DOI:** 10.3390/jcm10091890

**Published:** 2021-04-27

**Authors:** Gabriele Pesarini, Gabriele Venturi, Domenico Tavella, Leonardo Gottin, Mattia Lunardi, Elena Mirandola, Francesco Onorati, Giuseppe Faggian, Flavio Ribichini

**Affiliations:** 1 Division of Cardiology, Department of Medicine, University of Verona, 37126 Verona, Italy; gabriele.venturi.vr@gamil.com (G.V.); domenico.tavella@aovr.veneto.it (D.T.); dott.lunardim@gmail.com (M.L.); elenamirandola06@gmail.com (E.M.); flavio.ribichini@univr.it (F.R.); 2 Division of Anaesthesiology, University of Verona, 37126 Verona, Italy; leonardo.gottin@aovr.veneto.it; 3 Division of Cardiac Surgery, University of Verona, 37126 Verona, Italy; francesco.onorati@aovr.it (F.O.); giuseppe.faggian@aovr.it (G.F.)

**Keywords:** aortic valve stenosis, learning curves, CUSUM, quality-control, trans-catheter aortic valve implantation

## Abstract

Background: The aim of this research is to describe the performance over time of transcatheter aortic valve implantations (TAVIs) in a high-volume center with a contemporary, real-world population. Methods: Patients referred for TAVIs at the University Hospital of Verona were prospectively enrolled. By cumulative sum failures analysis (CUSUM), procedural-control curves for standardized combined endpoints—as defined by the Valve Academic Research Consortium-2 (VARC-2)—were calculated and analyzed over time. Acceptable and unacceptable limits were derived from recent studies on TAVI in intermediate and low-risk patients to fit the higher required standards for current indications. Results: A total of 910 patients were included. Baseline risk scores significantly reduced over time. Complete procedural control was obtained after approximately 125 and 190 cases for device success and early safety standardized combined endpoints, respectively. High risk patients (STS ≥ 8) had poorer outcomes, especially in terms of VARC-2 clinical efficacy, and required a higher case load to maintain in-control and proficient procedures. Clinically relevant single endpoints were all influenced by operator’s experience as well. Conclusions: Quality-control analysis for contemporary TAVI interventions based on standardized endpoints suggests the need for relevant operator’s experience to achieve and maintain optimal clinical results, especially in higher-risk subjects.

## 1. Introduction

Transcatheter aortic valve implantation (TAVI) represents an example of how the creative application of interventional concepts may translate into paradigm shifts for cardiovascular disease treatment. Nonetheless, a relevant issue linked to its expanding indications is the need for increased operator expertise and predictable immediate and long-term outcomes. In this view, it has been shown by large registries and clinical trials data that mastering the procedure requires a relevant learning curve that may be slightly simplified, but not completely flattened, by technical improvements [1,2,3,4,5]. Furthermore, more recent insights suggest that later-starting and more controlled TAVI programs may derive early outcome benefits by accurate center selection and rigorous proctoring by experts [6].

The aim of this work is to describe, using a dedicated statistical method, the procedural performance and the related clinical outcomes over time in a “real-life” population, after selecting the most appropriate valve type and implantation route, as per the Heart Team’s decision.

## 2. Materials and Methods

On an all-comer basis, patients who underwent TAVI at the University Hospital of Verona entered a prospective registry through the collection of complete baseline clinical, imaging, and biochemical features (Verona TAVI Registry). For the purpose of this work, all patients with a minimum follow-up of 30 days were considered. The type of valve and the access route (either transfemoral or transapical) were selected by the Heart Team. Balloon expandable Sapien devices (Edwards Lifesciences, Irvine, CA, USA) or Self-Expandable CoreValve prostheses (Medtronic Inc., Minneapolis, MN, USA) were used. Other brands’ prostheses were implanted infrequently in our center (<20 cases) and were therefore excluded from this analysis to avoid introducing incomplete learning curve biases. All the transfemoral procedures were performed by a single team of 2 interventional cardiologists (F.R. and G.P.), while all transapical procedures were carried out by one cardiac surgeon (F.O.) together with an interventional cardiologist (F.R. or G.P.) taking care of the actual valve positioning and deployment.

Procedural and follow-up data were entered into an electronic database, and relevant single events—together with standardized combined endpoints according to the Vascular Academic Research Consortium (VARC) 2 definitions—were analyzed [7].

In particular, device success is defined as the concomitant absence of procedural mortality, correct positioning of a single prosthetic heart valve into the proper anatomical location and intended performance of the prosthetic heart valve (no prosthesis–patient mismatch, mean aortic valve gradient < 20 mmHg or peak velocity < 3 m/s, and no moderate or severe prosthetic valve regurgitation).

Early safety at 30 days is defined as the concomitant absence of all-cause mortality, all stroke (disabling and nondisabling), life-threatening bleeding, acute kidney injury—Stage 2 or 3 (including renal replacement therapy), coronary artery obstruction requiring intervention, major vascular complication, and valve-related dysfunction requiring repeat procedure (balloon aortic valvuloplasty, TAVI, or SAVR).

Clinical efficacy after 30 days is defined as the absence of all-cause mortality, all stroke (disabling and nondisabling), hospitalizations for valve-related symptoms or worsening congestive heart failure, NYHA class III or IV, and valve-related dysfunction (mean aortic valve gradient > 20 mmHg, effective orifice area ≤ 0.9–1.1 cm^2^ and/or DVI < 0.35 m/s, moderate or severe prosthetic valve regurgitation).

VARC-2 standard definitions for death, stroke, bleeding, acute kidney injury (AKI), major vascular complications, and valve dysfunction were adopted [7]. All-cause vascular repair is defined as any form of action or intervention performed due to failed hemostasis or vessel failure after procedural closure with the selected hemostatic device(s).

Continuous data are reported as mean and standard deviation unless skewed, in which d median and interquartile range are provided. Categorical variables are expressed as numbers and proportions.

Cumulative sum analysis (CUSUM) was used to assess and illustrate the quality control of the procedures, as previously described [8,9]. Type I (α) and type II (β) errors were both set to 0.05. Acceptable and non-acceptable limits for upper and lower-boundary calculation were chosen according to the most relevant, recently published TAVI studies [2,3,4,5].

Single hierarchical endpoints reported in these studies and their online appendixes were considered to estimate the occurrence of VARC-2 endpoints in a “real world”, contemporary TAVI-population. For the device success composite endpoint, the CUSUM unacceptable limit was set to 10%, whereas the acceptable limit was set at 5%. Similarly, for the early safety composite endpoint, acceptable and unacceptable limits were selected at 10% and 20%, respectively. The procedure was defined as under control when the sum of failures curve laid between the calculated upper and lower boundary lines. Formal proficiency was defined as a better-than-expected performance of the equip at CUSUM analysis for the specific endpoint, characterized by the sum of failures curve lowering under the acceptable boundary reference line. The Society of Thoracic Surgeon (STS) score for mortality was considered to discriminate high (STS ≥ 8) or intermediate-low risk patients (STS < 8) [10].

The Verona TAVI registry data collection was approved by the local ethical committee and each patient provided written consent upon enrolment.

## 3. Results

After the exclusion of “other types of valves”, a total of 910 patients (46% males) with complete procedural and 30-days data underwent TAVI with either a CoreValve or a Sapien valve at the University Hospital of Verona between March 2010 and November 2020. Table 1 reports the baseline characteristics of the population.

**Figure 1:**
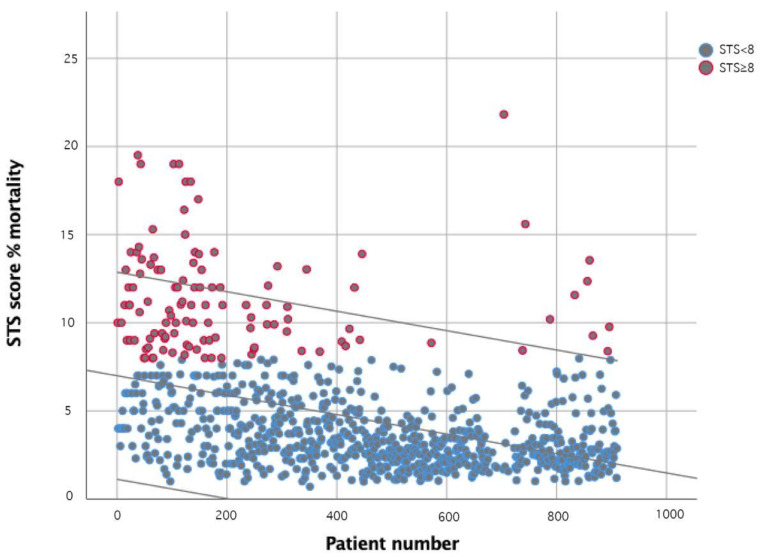
STS Score over time. Scatterplot with interpolation line, demonstrating the reduction of the patient’s risk as defined by STS score over time, from patients 1–910. Individual patients are represented by circles and divided into either a low-intermediate risk group (STS < 8) or high-risk group (STS ≥ 8).

The median age was 82 years, ranging from 24 to 97 years. Median Logistic Euroscore, Euroscore II, and STS-Score were 15.3 [16.4]%, 4.6 [5.3]%, and 3.5 [3.5]%, respectively. One hundred and fifteen patients (12.6%) presented with an STS-Score ≥ 8%. A scatterplot of the risk level, expressed as an STS score for mortality in subsequent patients, is depicted in Figure 1.

Of note, and as expected, STS for mortality decreased continuously during the enrollment, passing from a median of 7% at the beginning to a median of 2.4% for the last cases. The majority of subjects had some degree of renal impairment: in fact, for 67.4% of patients, eGFR was <60 mL/min/m^2^, while it was <30 mL/min/m^2^ in 16.8%. The overall ejection fraction median was 55%, while it was reduced to under 50% in 20.4% of cases.

As far as the prosthesis types are concerned, we implanted 608 patients (66.8%) with balloon-expanded Edwards prosteses. Specifically, we implanted 28 patients with the original Sapien device and 100 patients with the Sapien XT valve in the first part of the experience. Later, we implanted the more recent Sapien3 (*n* = 354) and Sapien3 Ultra devices (*n* = 126). Of these patients, 156 subjects were treated within the cath lab facility via the transapical route in equip by dedicated cardiac surgeons.

For the self-expandable implants, we treated a total of 302 patients (33.2%) with the Medtronic Platform. Specifically, 49 patients were trated with the CoreValve device, 181 with the Evolut R, and finally, 72 patients with the Evolut Pro prosthesis.

Detailed procedural and 30-day events are reported in Table 2, according to the level of risk.

There were 3 procedural deaths (0.3%) and 18 total deaths at 30 days, of which 9 were of cardiovascular nature (1.0%) and mainly clustered in the high-risk subgroup (7.0 vs 1.2%, *p* = 0.04). In total, two of the three procedural deaths occurred within the first 15 cases of transapical procedures. There were seven cases of stroke in total (major and minor), of which three were disabling (0.4%), while only two cases of acute relevant coronary obstruction occurred intra-procedurally or immediately after procedure (one transfemoral and one transapical). Suboptimal positioning requiring a second valve implantation occurred in 12 (1.3%) cases (one transapical), while suboptimal prosthesis performance at post-procedural echocardiogram occurred in 30 cases (3.3%).

Events related to the vascular access that occurred more frequently were major vascular complications in 27 patients (3.3%) and life-threatening bleedings in 14 (1.5%).

Combined VARC-2 endpoints were therefore evaluated and quantified as follows: Early safety events occurred in 87 patients (9.5%) and mainly, even if not significantly, in the high-risk group (12.2 vs. 9.2%; *p* = 0.3); device success was not achieved in 43 patients (4.7%)—5.2% high risk vs. 4.7% intermediate-low risk (*p* = 0.79). Only data relative to the trans-femoral procedures were comparable. STS high-risk group classification was homogeneous between balloon-expandable (13.5%) and self-expanding prostheses (10.9%; *p* = 0.3). Lack of device success was also similar in both valve types (4.1 vs. 5.9%; *p* = 0.23), as well as the early safety endpoint (9.6% vs. 9.5%; *p* = 0.98).

At complete follow-up, clinical efficacy endpoint was reached in 77.4% of subjects, without differences between the two valve types. As expected, however, 61 patients in the high-risk STS group (53.0%) versus 145 in the lower STS group (18.2%) did not attain the VARC2 clinical efficacy (*p* < 0.001) goal.

### 3.1. CUSUM Analysis for VARC-2 Device Success Endpoint

**Figure 2:**
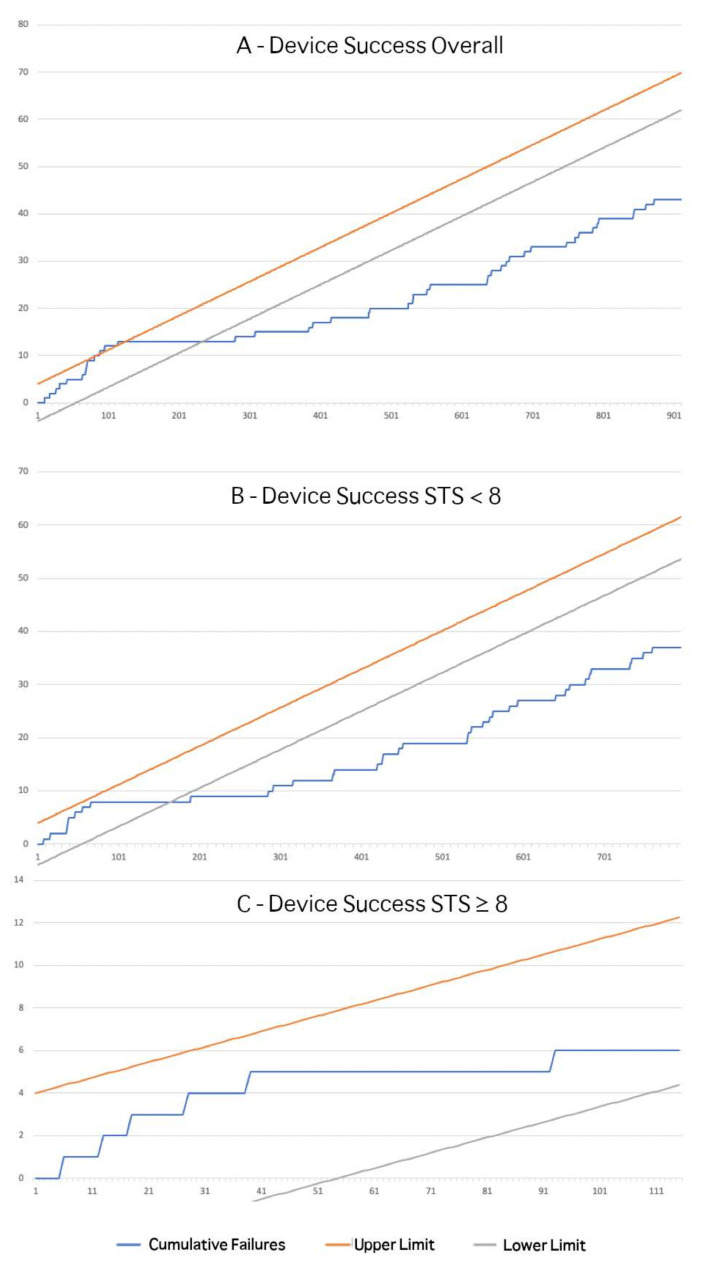
CUSUM Analysis for device success. Incremental line represents unadjusted cumulative sum of failures. Lower and upper limits are calculated upon acceptable and unacceptable failure rates of 5% and 10%, respectively. Observations above the upper limit indicate out of control procedure, while observation below the lower limit suggest better-than-expected results (proficiency). CUSUM analyses are provided for the overall population (**A**), low-intermediate risk (**B**), and high-risk subgroups (**C**).

As clearly depicted by the curve in Figure 2A, an early learning curve is evident for cases from 1 up to 126, while afterwards the TAVI intervention starts to remain permanently under control, with the team performing better than reported in trials (proficiency) for this endpoint after 230 cases.

The effect of the patient’s basal risk is explained by the Figure 2B,C, where a flattening CUSUM curve can be appreciated after 35 cases for patients with STS scores < 8 (Figure 2B), with formal proficiency reached and maintained after 150 cases. Procedures performed in higher risk patients (Figure 2C) proved always under control within the expected boundaries but never attained better-than-expected results (proficiency) within this series. If transapical cases only were considered, a similar curve showing an always-in-control procedure can be observed, with formal proficiency reached after 110 cases.

### 3.2. CUSUM Analysis for VARC-2 Early Safety Composite Endpoint

**Figure 3:**
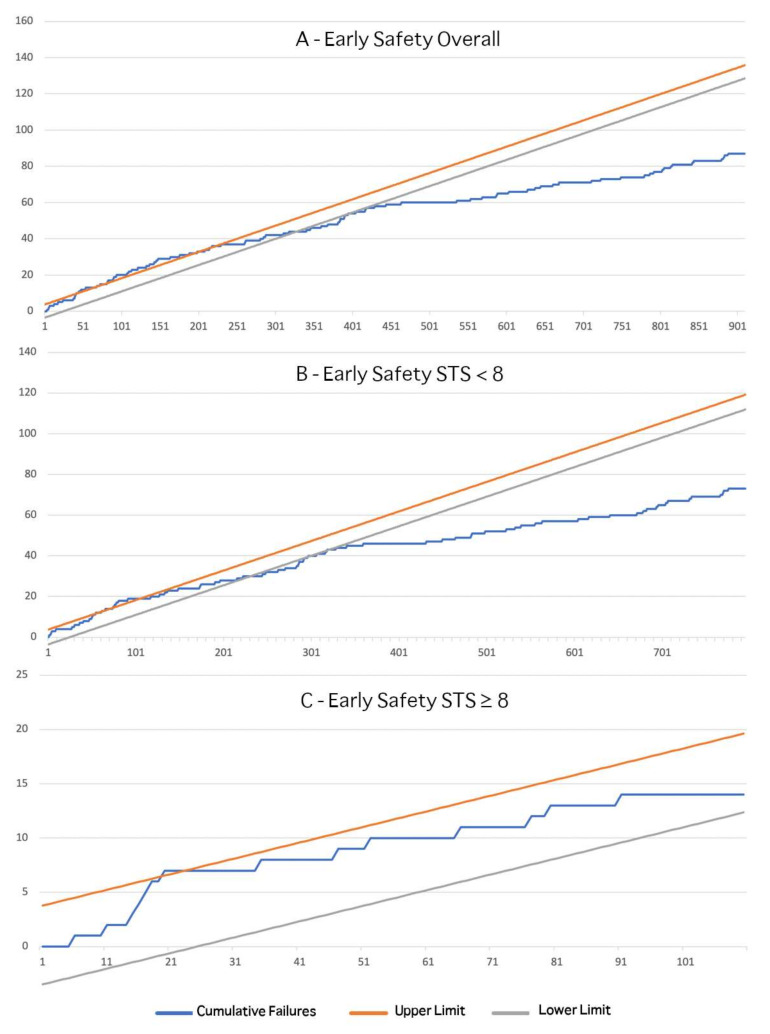
CUSUM Analysis for early safety. Incremental line represents unadjusted cumulative sum of failures. Lower and upper limits are calculated upon acceptable and unacceptable failure rates of 10 and 20%, respectively. Observations above the upper limit indicate out of control procedure, while observation below the lower limit suggest better-than-expected results (proficiency). CUSUM analyses are provided for the overall population (**A**), low-intermediate risk (**B**) and high-risk subgroups (**C**).

As expected, the initial learning curve for this endpoint was more challenging overall, with the first 190 procedures sitting at the edge of the acceptable upper boundary, representing a procedure not always “under control” when the contemporary, more conservative limits are applied to the initial TAVI patient population (Figure 3A).

The cumulative events curve flattens afterwards, suggesting formal proficiency after 320 cases were performed by the team, with clinical results comparing favorably to those reported in trials. Again, the baseline risk level played a role, with “borderline” outcomes until case n° 78 for patients with an STS score ≥ 8 (Figure 3C), a population that never lowered the safety endpoint below the proficiency boundary. Therefore, in our experience, given their high-risk clinical profile, these patients struggled to achieve the clinical results obtained in intermediate and low-risk subjects enrolled in recent clinical trials, even in a highly experienced center.

**Figure 4:**
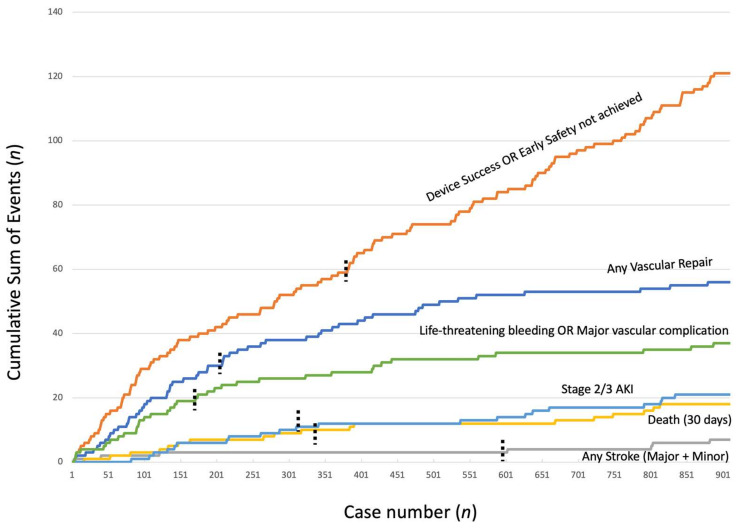
Individual endpoints analysis. Cumulative occurrence of the individual, more clinically relevant VARC-2 endpoints is provided. Vertical dashed bars represent the point at which 50% of each event had occurred.

Figure 4 depicts the cumulative occurrence of the most relevant procedure-related endpoints. Vertical dashed lines represent the temporal point of occurrence of 50% of the specific event. Apart from disabling stroke, which occurred in very few cases, half of the occurrence of 30 days mortality, major vascular complications or life-threatening bleedings, AKI stage 2/3 and need for vascular repair were clustered in the first third of cases. However, subsequent flattening of the event curves was quite slow, suggesting diluted but relevant endpoint occurrence even in the advanced phase of the procedural experience. Furthermore, when considering failure to achieve VARC2 early safety or device success, half of the events occurred after case 385 (43%) of the entire case load, thus confirming the presence of a relevant number of combined events even in the advanced phase of the center’s experience.

## 4. Discussion

The evolution of TAVI materials and the standardization of the procedure have ensured high technical success rates and more user-friendly platforms. Importantly, over the years, good clinical results and low incidence of valve dysfunction have been demonstrated since the initial experiences in high-risk patients [11,12,13,14]. However, the accepted complications and unsuccess at the beginning of the TAVI experience were set on the dismal prognosis of the patients that were initially treated, i.e., inoperable or extremely high risk [1]. More recently, the interest in treatment of intermediate and low-risk patients has been justified by dedicated randomized clinical trials [4,5] and, as a consequence, the median baseline risk level of real-world subjects referred for TAVI by the Heart Teams is clearly decreasing. In this field of lower-risk subjects, conventional surgery has a proven history of efficacy and safety, and the cost-benefit ratio of percutaneous procedures is still debated. Therefore, even if better knowledge of the procedures and newer materials may advantage centers starting their experience, higher interventional standards and lower rates of acceptable complications become imperative before dealing with this rapidly changing clinical landscape.

In this work, we describe the incidence rates of major periprocedural events and compared them to the results derived from the latest international TAVI trials that enrolled intermediate and low-risk patients [2,3,4,5].

As illustrated in Figure 1, risk level as defined by STS score steadily and significantly decreased over time. However, even in the last part of the experience, higher-risk and outlier patients did not disappear, as a concrete legacy of the original TAVI target population. Therefore, the goal of a futureproof TAVI-team is twofold: reaching and maintaining excellent procedural and clinical results to justify the treatment of intermediate and low-risk patients, while keeping proficiency in treating complex, high-risk subjects and dealing with their possible multilevel complications. In this view, centralization of TAVI in heart valves centers with established heart teams and large volumes of patients may represent the more logical option for patient referral and spoke-center operator training and integration in a well-coordinated, quality-oriented environment.

Single endpoints at 30 days, in our experience, fairly matched those reported in landmark studies in terms of cardiovascular mortality (1.0% vs. 0.4–2.9%), stroke (0.8% vs. 0.5–3.2%), and major vascular complications (3.0% vs. 2.2–7.9%) [4,5,15]. Of note, only a few publications report the rates for the VARC-2 standardized composite endpoints, limiting the comparability of literature data. Our study defines contemporary acceptable and unacceptable limits of VARC-2 procedural success (5–10%) and early safety (10–20%) endpoints to facilitate procedural quality analysis, extrapolation, and comparison.

In the early TAVI days, the outcome of a relatively small number of cases correlated better technical performances in high-risk patients through a simple chi square test that compared major clinical events observed before and after the performance of 25–30 consecutive cases [16]. The application of the most appropriated CUSUM statistical method to the TAVI, as performed in our initial experience with mostly high-risk patients, better defined this rudimentary learning curve observation [8]. Indeed, by applying the acceptable clinical boundaries—defined by the randomized clinical trials performed at that time in higher-risk TAVI patients—the CUSUM analysis revealed that a minimum of 54 and 32 patients were required to warrant acceptable early safety and device success, respectively, as defined by the same VARC-2 endpoints.

Despite the widespread use of TAVI in clinical practice worldwide in the last 10 years, procedural performance based on the widely accepted VARC-2 endpoints is still rarely reported. Only recently has a large registry, based on more than 61,000 patients treated with balloon-expandable valves and using comparable endpoints, suggested initial learning curves of up to 200 cases [17]. Additionally, it has been demonstrated that operator [18] and institutional [19] experience is linked to outcomes after TAVI, showing also that low-volume centers (<50 procedures/year) expose patients to reduced procedural safety and higher mortality, a compelling observation that stresses the need for the concentration of patient referral to dedicated, high-volume heart valve centers [20]. Supporting this critical concept, an interesting analysis performed on 113,500 patients treated with TAVI in the US between 2015 and 2017 confirms an inverse association with mortality of both center and single-operator case volume [21]. However, no dedicated methods such as CUSUM were applied to analyze procedural control in these large sample studies.

The main purpose of our work is to describe the procedural quality analysis of a single TAVI team, targeting the clinical results expected for contemporary patients as indicated by randomized clinical trials that assessed the measurable and comparable VARC-2 endpoints.

Our present findings confirm, and further expand, the relevant figures for the initial TAVI learning curve, as previously reported regarding our group in a high-risk population [8]. As expected, even more experience is needed to satisfy the stringent current boundaries set for both device success and early safety endpoints in a team that starts treating a contemporary (intermediate risk) TAVI population. Indeed, about 125 and 190 consecutive cases are needed, respectively, to align the procedural quality to the results reported by latest trials [2,3,4,5] (Figure 2 and Figure 3) in terms of VARC-2 device success and early safety, respectively.

In addition, especially for clinical efficacy and early safety, the required experience to achieve proficient results increases with patients’ baseline risk, supporting the idea to centralize TAVI procedures in high-volume “heart valve” centers, primarily for complex patients. In fact, given the demanding nature of TAVI in terms of human, organizational, and economic resources, an effective TAVI (heart) team should cautiously take into account the clinical needs of the referring region, continuously monitor the outcomes to optimize their investment, and prevent dispersion of health-system resources due to sub-optimal cost-benefit balances. Of note, in our sample, procedures in patients with STS scores equal to or higher than 8 points proved in-control through the observation. However, in this particular subgroup, formal proficiency was never reached, a behavior that may be related to the relatively low proportion of high-risk subjects (12.6% overall) coupled with the more stringent acceptable and unacceptable event rates that we derived from intermediate/low-risk trials. Nevertheless, these patients deserve special care to receive intervention in more stable conditions, in order to permit minimally invasive management and accurate positioning of the device for maximum device success and to avoid procedure-related complications. Furthermore, especially for early safety, we found a significant increase in the rate of all-cause 30-days deaths (7.0% vs. 1.2%), which may reinforce the need for careful heart-team patient selection and expert peri and post-procedural management.

Finally, in analyzing the cumulative occurrence of clinically relevant single endpoints, a sustained reduction with experience was clearly detected for all of them. However, even though there was a clustering in the first third of cases for adverse procedural events, especially vascular complications and need for repair, they continued to occur over time despite experience and a reduced proportion of high-risk patients. These may be more linked to the peculiar characteristics of each patient, rather than to the contemporary interventional technique or improved materials, but it is very likely that a center’s experience may limit their impact on a patient’s final outcome.

### Study Limitations

When analyzing a single-team performance, the learning curve should be unaffected by operator changes and therefore representative of an initial “naïve” experience; however, specific center features may limit the generalizability of the conclusions compared to multicenter registries. Furthermore, even though the team in our study worked together at all times, the adoption of two types of valves (balloon expandable and self-expanding) and two access routes (transfemoral and transapical) may add variables to the learning curves, although the differences observed between types of access or prostheses were not significant.

## 5. Conclusions

Quality-control analysis for real-life TAVI procedures based on standardized VARC-2 composite endpoints suggests that a relevant experience (up to 190 cases) is needed to achieve clinical results comparable with those of the latest intermediate and low-risk patient randomized trials. Furthermore, the occurrence of clinically relevant single endpoints appears to be influenced by experience in most cases. Higher STS scores define a subgroup of patients with poorer mid to long-term outcomes and may require even more experienced operators.

## Figures and Tables

**Table 1:** Baseline characteristics of study population.

Parameter	Total Sample *n* = 910	STS ≥ 8 *n* = 115 (12.6%)	STS < 8 *n* = 795 (87.4%)	*p*
Age, years	82 (24–97)	83 (52–91)	81 (64–89)	0.001
Logistic EuroSCORE, %	15.3 (16.4)	28.0 (21.8)	13.8 (14.0)	<0.001
EuroSCORE II, %	4.6 (5.3)	11.0 (8.4)	4.0 (3.9)	0.002
STS score, %	3.5 (3.5)	10.5 (2.4)	3.2 (2.3)	<0.001
Male, *n* (%)	419 (46.0%)	53 (46.1%)	366 (46.0%)	0.99
BMI, kg/m^2^	25.4 (5.7)	24.1 (5.5)	25.5 (5.7)	0.23
eGFR (mL/min/1.72 m^2^)	47.7 (28.1)	35.1 (19.2)	50.1 (28.3)	<0.001
Anemia, *n* (%)	455 (50.0%)	65 (56.5%)	390 (49.2%)	0.14
Dyslipidaemia, *n* (%)	506 (55.6%)	78 (67.8%)	428 (55.0%)	0.01
COPD, *n* (%)	149 (16.4%)	38 (33.9%)	111 (14.3%)	<0.001
Diabetes, *n* (%)	255 (28.0%)	46 (40.4%)	209 (26.9%)	0.003
Hypertension, *n* (%)	766 (84.2%)	103 (90.4%)	663 (85.1%)	0.13
Previous AMI, *n* (%)	136 (14.9%)	26 (23.0%)	110 (14.2%)	0.01
Atrial fibrillation, *n* (%)	343 (37.7%)	49 (42.6%)	294 (37.9%)	0.34
Previous stroke, *n* (%)	65 (7.1%)	12 (10.4%)	53 (6.8%)	0.16
PVD, *n* (%)	302 (33.2%)	64 (55.7%)	238 (30.6%)	<0.001
CAD, *n* (%)	418 (45.9%)	74 (64.3%)	344 (42.9%)	<0.001
Previous CABG, *n* (%)	94 (10.3%)	19 (16.5%)	75 (9.6%)	0.025
Previous AVR, *n* (%)	59 (6.5%)	7 (6.1%)	52 (6.7%)	0.81
Previous MVR, *n* (%)	47 (5.2%)	6 (5.2%)	41 (5.3%)	0.99
PM, *n* (%)	109 (12.0%)	16 (13.9%)	93 (11.7%)	0.49
Syncope, *n* (%)	163 (17.9%)	33 (28.7%)	130 (16.7%)	0.002
Stable angina, *n* (%)	157 (17.3%)	42 (36.5%)	115 (14.7%)	<0.001
Unstable angina, *n* (%)	43 (4.7%)	12 (10.4%)	31 (4.0%)	0.002
Cardiogenic shock or APE, *n* (%)	121 (13.3%)	49 (42.6%)	72 (9.2%)	<0.001
NYHA class I or II, *n* (%)	239 (26.2%)	16 (13.9%)	223 (30.3%)	<0.001
NYHA class III or IV, *n* (%)	671 (73.8%)	99 (86.0%)	572 (71.9%)	<0.001
Prior PCI, *n* (%)	143 (15.7%)	30 (26.1%)	113 (14.2%)	0.001

BMI: body mass index; eGFR: estimated glomerular filtration rate; COPD: chronic obstructive pulmonary disease; PVD: peripheral vascular disease; CAD: coronary artery disease; CABG: coronary artery bypass grafting; AVR: aortic valve replacement; MVR: mitral valve replacement; PM: pacemaker; APE: acute pulmonary edema; NYHA: New York Heart Association; PCI: percutaneous coronary intervention. Categorical data are presented as numbers and percentages; continuous data are presented as means ± standard deviations for normally distributed variables, and as median and interquartile range otherwise. Age is presented as mean and range (min-max).

**Table 2:** Thirty-day adverse events according to VARC2.

Variables	All Patients *n* = 910	Group STS ≥ 8, *n* = 115 (12.6%)	Group STS < 8, *n* = 795 (87.4%)	*p*
Device success not achieved, n (%)	43 (4.7%)	6 (5.2%)	37 (4.7%)	0.79
Procedural mortality, *n* (%)	3 (0.3%)	1 (0.9%)	2 (0.4%)	0.77
Suboptimal positioning, *n* (%)	12 (1.3%)	4 (3.5%)	8 (1.0%)	0.24
Non-Intended performance of the prosthetic heart valve, *n* (%)	30 (3.3%)	3 (2.6%)	27 (3.4%)	0.89
Early safety not achieved (30 days), *n* (%)	87 (9.5%)	14 (12.2%)	73 (9.2%)	0.30
All-cause mortality, *n* (%)	18 (2.0%)	8 (7.0%)	10 (1.2%)	0.03
Cardiovascular, *n* (%)	9 (1.0%)	4 (3.5%)	5 (0.6%)	0.03
Non cardiovascular, *n* (%)	9 (1.0%)	4 (3.5%)	5 (0.6%)	0.01
All stroke, *n* (%)	7 (0.8%)	2 (1.7%)	5 (0.6%)	0.07
Life-threatening bleeding, *n* (%)	14 (1.5%)	2 (1.7%)	12 (1.5%)	0.97
Acute Kidney Injury stage 2 or 3, *n* (%)	21 (2.3%)	3 (2.6%)	18 (2.3%)	0.73
Coronary artery obstruction requiring intervention, *n* (%)	2 (0.2%)	0 (0%)	2 (0.3%)	0.65
Major vascular complications, *n* (%)	27 (3.0%)	4 (3.5%)	23 (2.9%)	0.46
Valve-related dysfunction requiring repeat procedure, *n* (%)	9 (1.0%)	5 (4.3%)	4 (0.5%)	0.08
Clinical efficacy not achieved, *n* (%)	206 (22.6%)	61 (53.0%)	145 (18.2%)	<0.001

## Data Availability

The data presented in this study are available on request from the corresponding author. The data are not publicly available due to local policy regulating patients’ data sharing.
